# Perspectives of general dental practitioners on preventive, patient-centred, and evidence-based oral healthcare—A Q-methodology study

**DOI:** 10.1371/journal.pone.0219931

**Published:** 2019-08-20

**Authors:** Fatiha Baâdoudi, Job N. A. van Exel, Fatima M. Ali, Neal Maskrey, Geert J. M. G. van der Heijden, Denise Duijster

**Affiliations:** 1 Department of Social Dentistry, Academic Centre for Dentistry Amsterdam, University of Amsterdam and VU University, Gustav Mahlerlaan, Amsterdam, The Netherlands; 2 Erasmus School of Health Policy & Management, Erasmus University Rotterdam, Rotterdam, The Netherlands; 3 School of Pharmacy, Keele University, Newcastle under Lyme, Staffordshire, United Kingdom; Western Sydney University, AUSTRALIA

## Abstract

**Objective:**

In the last 30 years, innovations in oral healthcare (OHC), such as advanced restorative techniques, shifts towards preventive and evidence-based care and changes in patients’ expectations, have increased the complexity of clinical decision-making in OHC. Little is known about the perspectives of general dental practitioners (GDPs) on the value of providing preventive, patient-centred and evidence-based OHC. This study aimed to explore the range of perspectives present amongst GDPs on OHC.

**Method:**

Q-methodology was used to explore perspectives among 78 GDPs working in the Netherlands. Participants were asked to sort 50 statements representing three central domains in OHC: i.) restorative versus preventative OHC, ii.) disease-centred versus patient-centred OHC and iii.) expertise-based versus evidence-based OHC. Opinion statements about delivering OHC were formulated on the basis of published literature and input from OHC professionals. By-person factor analysis was used to reveal clusters of communality in statement rankings, which were interpreted and formed perspectives on OHC.

**Results:**

Four perspectives, explaining 47% of variance, on OHC were identified amongst GDPs: ‘the patient-focused dentist who values prevention’, ‘the outcome-oriented dentist who values learning from colleagues’, ‘the team player with ultimate care responsibility’ and ‘the dentist who considers oral health the responsibility of the patient.’

**Conclusion:**

Q-methodology can be effectively used to describe the different perspectives that GDPs have on the challenges of preventive, patient-centred and evidence-based OHC. GDPs should not be seen as a homogenous group; rather they have different views and approaches to the care they provide. This has implications for health systems; awareness of the heterogeneity of practitioners’ perspectives can potentially be used to develop bespoke quality of care improvement strategies that constructively engage with each of these different groups.

## Introduction

Dentistry has been in a period of rapid change—as reflected in technical developments, higher patient expectations and a shift towards both preventive and evidence-based care [1]. Development of advanced restorative materials and techniques have broadened the possibilities and consequently the expectations of oral healthcare (OHC). Due to more availability of aesthetic treatment approaches, patients expectations have not only increased for functionality but also for good-looking teeth [2]. Increasingly, patients are critical consumers that value explicit demonstration of competence of oral health professionals. Further, prevention and evidence-based care have become key concepts for improving the quality of oral health and OHC. Simultaneously, variation in the delivery and quality of OHC has become explicit, leading to a growing interest in identifying and reducing unexplained variation in OHC [3].

Hence, many decisions about OHC are now preference-sensitive [4]. General dental practitioners (GDPs) obviously have different perspectives about the value of preventive, patient-centred and evidence-based care, which could contribute to variation in the delivery and quality of OHC. However no studies have been performed to explore these perspectives amongst GDPs. Some GDPs may see their role as primarily technical, whereas others may approach their role from a wider, additional perspective as a health adviser. Some patients may see their dentist in a purely technical, restorative role, while others may seek a preventative and aesthetic focus from their carer. For example, implantology and other new materials and techniques have widened the options in restorative consultations, whilst in those same consultations a range of oral health preventative interventions and advice may also be both indicated and needed. Which takes precedence? Which are chosen? How should GDPs respond to these challenges in delivering OHC? It seems unlikely that all GDPs would respond in the same way. Such variation, if unwarranted, might indicate that the care provided is not optimally serving the needs and preferences of patients. It might also illustrate an opportunity to reduce costs and increase the quality of care delivery without compromising patient care.

Bertakis et al. [5] and Jefferson et al. [6] showed variation in care delivery in the length of communication and consultation with patients according to the gender of GDPs. Variation in OHC delivery may depend, among many other factors, on the views GDPs hold regarding their own professional role and preferences for certain dental treatments. Yet, little more is known about the perspectives of GDPs on key aspects of OHC—their perceptions of the relative importance of restorative techniques, prevention, evidence-based practice and patient involvement. Exploring GDPs’ conscious and unconscious preferences and attitudes may provide further insights into the observed variation in care delivery. Understanding the perspectives of GDPs could be used to inform more targeted quality improvement strategies aiming to further develop and extend the role of dental professionals, which are aligned with the potentially diverse needs of GDPs. For individual practitioners, self-awareness of their own preferences and attitudes may help GDPs to better match their clinical expertise and knowledge of the evidence-base for their advice and treatments with the values and preferences of patients, especially when their own values and preferences may be very different from those held by individual patients. Therefore, this study aimed to explore the range of perspectives on preventive, patient-centred and evidence-based OHC among GDPs in the Netherlands using Q-methodology.

## Methods

Q-methodology combines qualitative and quantitative techniques to explore subjective phenomena [7–10]. In a Q-methodology study, purposively selected respondents are presented with a set of statements about the study topic. Purposive sampling is used to include a diverse group of GDPs, according to characteristics that are anticipated to relate to different perspectives. Respondents are instructed to rank these statements according to agreement or importance using a sorting grid. Following this, respondents are asked to explain their ranking, with emphasis on the statements placed at the extreme ends of the grid. By-person factor analysis is used to identify groups of respondents that ranked the statements in a similar way. The underlying assumption is that participants reveal their view on the topic through ranking the statements, and that respondents with similar rankings therefore have similar views. The factors resulting from the analysis together with the qualitative data obtained in the interviews are interpreted and described as shared perspectives on the topic of study [9].

### Participants and study design

The study was conducted among GDPs in the Netherlands. They were purposively selected according to gender, age, region and practice composition (group or solo). These characteristics were considered potentially important in relation to their view on oral health care, and therefore GDPs were recruited until all these characteristics were sufficiently represented. This was in order to minimize the risk of missing participants with a less prevalent viewpoint on OHC. Participants were recruited from the Royal Dutch Dental Association (KNMT) database, the largest dental association in the Netherlands. The KNMT supports IQual groups where groups of GDPs meet on a monthly basis for in-service training and education. IQual groups facilitate sharing knowledge and experience in a safe and open environment. GDPs were invited to participate in the study by means of an email sent out using the IQual register database. Additional recruitment took place after short presentations to inform the GDPs about the study during IQual conferences of the KNMT. In addition, GDPs were recruited from the research team’s network using a snowballing approach. GDPs were free to participate individually or as an IQual group. From the GDPs that applied to participate in the study, a purposive sampling was made to ensure variation in gender, age, region and practice composition. There is no definite number of participants required in a Q-methodology study; in general between 40 and 60 participants is considered to be adequate [9]. Data collection started in May 2017 and finished in December 2017.

### Statement set development

The statement set was developed using a structured approach in order to assure that the set was broadly representative of all aspects that are relevant to OHC delivery. The statements were based on recent research [11–13], and incorporated expertise within the research team at the Academic Centre for Dentistry Amsterdam (ACTA). Three key domains characterizing OHC delivery were identified from the literature: i.) restorative—preventative OHC, ii.) disease-centred—patient-centred OHC and iii.) expertise-based—evidence-based OHC. Next, three different sources of information were used to define the initial statement set: i.) published literature on attitudes of GDPs [14,15]; ii) input from six academically working GDPs from ACTA that each provided two to three statements per domain based on their professional expertise; and, iii) interviews with eight individual OHC professionals (e.g. researchers, teachers, dental hygienists). The resulting 199 statements were reviewed by two researchers (FB and DD) independently. In an iterative process, statements were categorized according to the three domains, sorted into coherent themes within each domain, and any duplicates were removed. Next, between one and three statements were selected to represent each theme, and some statements were edited to improve their readability. Finally, a third researcher (JvE) reviewed the procedure and inspected the statement set for comprehensiveness and clarity.

To test the comprehensiveness and clarity of the statement set among GDPs the set was then piloted with four GDPs. Statements which those GDPs considered missing but important were added in the final statements set, resulting in a final set of 50 statements. S1 Table presents the full list of statements, categorized according to themes and domains.

### Study procedure

GDPs willing to participate as a group were interviewed by a researcher during their IQual meeting. Mutually convenient appointments were made with the individual GDPs who were recruited. Before the interview, an information letter was sent to the participants. At the start of the interview, participants received a short introduction of the study aim and procedure, and they were asked to sign an informed consent form. GDPs participating as a group were instructed to do the exercise individually, without discussion with other group members. Each participant was presented with the 50 statements printed on cards. They were asked to read all statements, and to sort them into three piles: statements they agreed with, statements they were neutral about and statements they disagreed with. Following this, participants were asked to rank the statements on a sorting grid ranging from most agree (score +5) to least agree (score -5) printed on an A1-sheet that had been placed before them. Each statement had to be placed in one of the blank cells on the grid based on their strength of agreement with the statement. After finishing the ranking exercise, participants were encouraged to rearrange the statements on the grid until they were satisfied with the full sort, after which they were asked to populate a paper copy of the sorting grid using the unique numbers on each statement. Next, each participant was asked to elaborate on the statements they placed at the extreme ends of the sorting grid, the two statements they least agreed with and the two statements they most agreed with, and to describe their view about their profession in two or three sentences. Finally, participants were asked to fill in a short demographic questionnaire, including age, gender, practice composition, year of graduation and region. Collected data were anonymized to preclude the possibility of re-identification.

### Data analysis

Q-methodology differs from most other approaches that use factor or cluster analysis to identify groupings in the data in that it examines the correlation among people rather than the items, as it seeks to identify patterns of diversity and similarity between people [9]. The purpose is to identify the range of perspectives in a population and not the proportion of the population that holds these perspectives, and therefore a comparatively small, selected sample of the population is sufficient [[16,17]].

The assumption underlying the analysis is that respondents who, with the same instruction, ranked the set of statements in a similar way, have a similar view on the topic [9]. Therefore, the collected rankings of the statements were correlated and subjected to by-person factor analysis (i.e., centroid factor extraction with varimax rotation) using PQmethod 2.35 software [18].

Factor solutions consisting of factors with Eigenvalue larger than 1 and at least two respondents defining each factor (p < 0.05) were inspected. For each factor in the selected factor solution, a weighted average ranking of the statements was calculated, based on the rankings of the respondents defining the factor and their correlations with the factor as weights. Next, each factor was interpreted as a perspective on OHC using this idealized sort of the statements, with specific attention to the characterizing statements, i.e. those ranked at the extreme ends of the sort for that factor, and the distinguishing statements, i.e. those ranked statistically significantly differently (p < 0.05) than in the factor as compared to the other factors. Finally, the qualitative data supplied by respondents were used to verify the factor interpretations, and some citations were extracted and included in the description of the factors as illustration.

## Results

**Fig 1:**
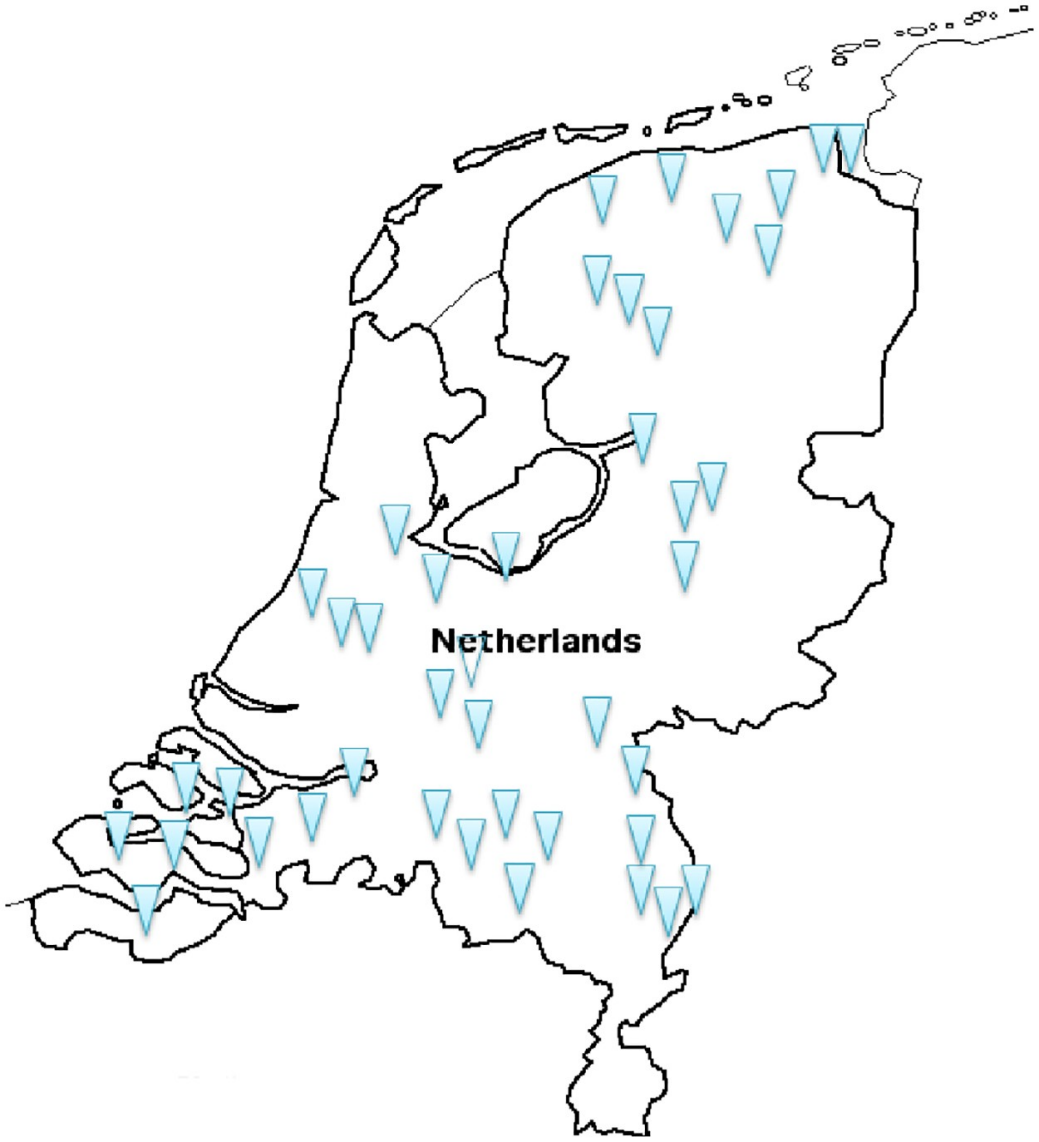
The location of dental practices of the participating GDPs.

A total of 79 participants from 41 dental practices participated in this study. One GDP was excluded from the study because there were more than two missing statements in the paper copy of the sorting grid. The final sample consisted of 78 GDPs of whom 53 (69%) were male, 24 (31%) were female and 1 preferred not to reveal gender. The mean age of the GDPs was 51 years with a range of 26 to 69 years, thus covering the full career age span. Table 1 shows the demographic characteristics of the study participants, and Fig 1 displays the regional distribution of the dental practices.

**Table 1:** Q-sample characteristics.

Characteristics (n = 78)		Dentists n (%)
Gender		
	Male	53 (69)
	Female	24 (31)
Age		
	26–40	16 (21)
	41–55	27 (36)
	56–69	33 (43)
Practice*		
	Group	49 (63)
	Solo	33 (42)
Years since graduation		
	0–15	8 (11)
	16–30	27 (39)
	31–45	35 (50)
Works regularly with a dental hygienist or prevention assistant		
	Within the practice	48 (72)
	External	14 (21)
	No	5 (7)

*GDPs can work in both group and solo practices.

**Fig 2:**
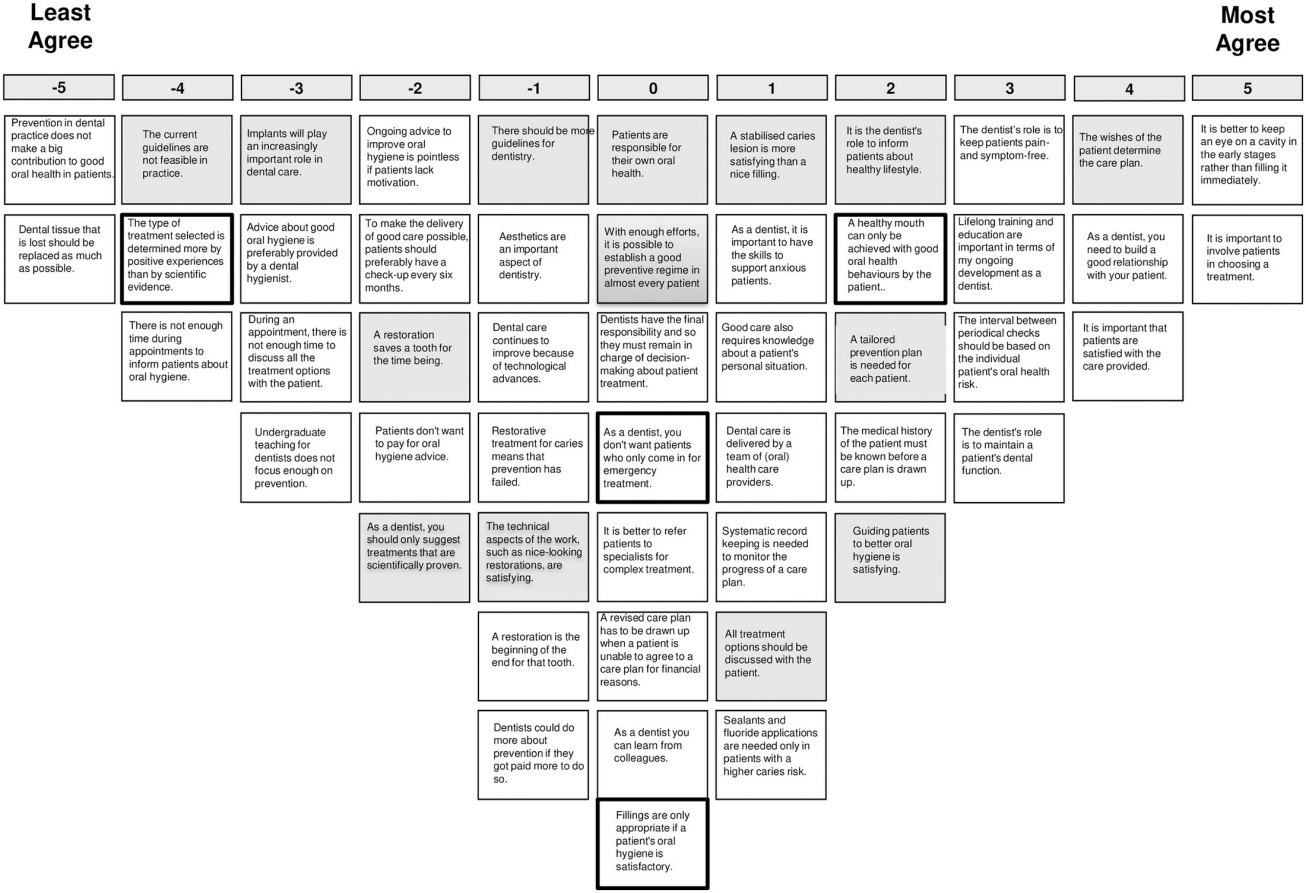
Score sheet for viewpoint 1. Note: Distinguishing statements (p<0.05) are shown in black outlined boxes, distinguishing statements (p<0.001) are shown in grey boxes.

**Fig 3:**
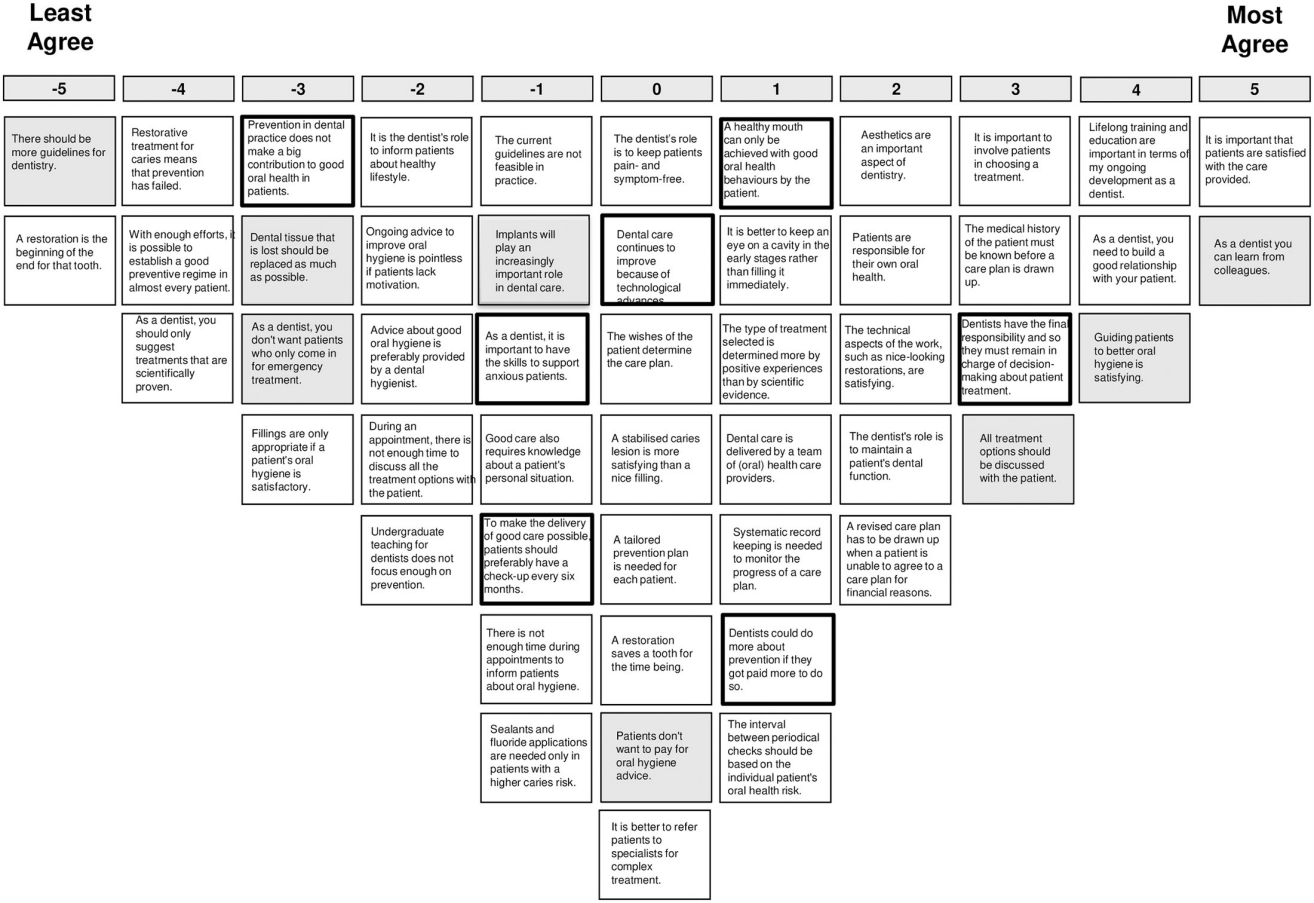
Score sheet for viewpoint 2. Note: Distinguishing statements p<0.05 are shown in black outlined boxes, distinguishing statements p<0.001 are shown in grey boxes.

**Fig 4:**
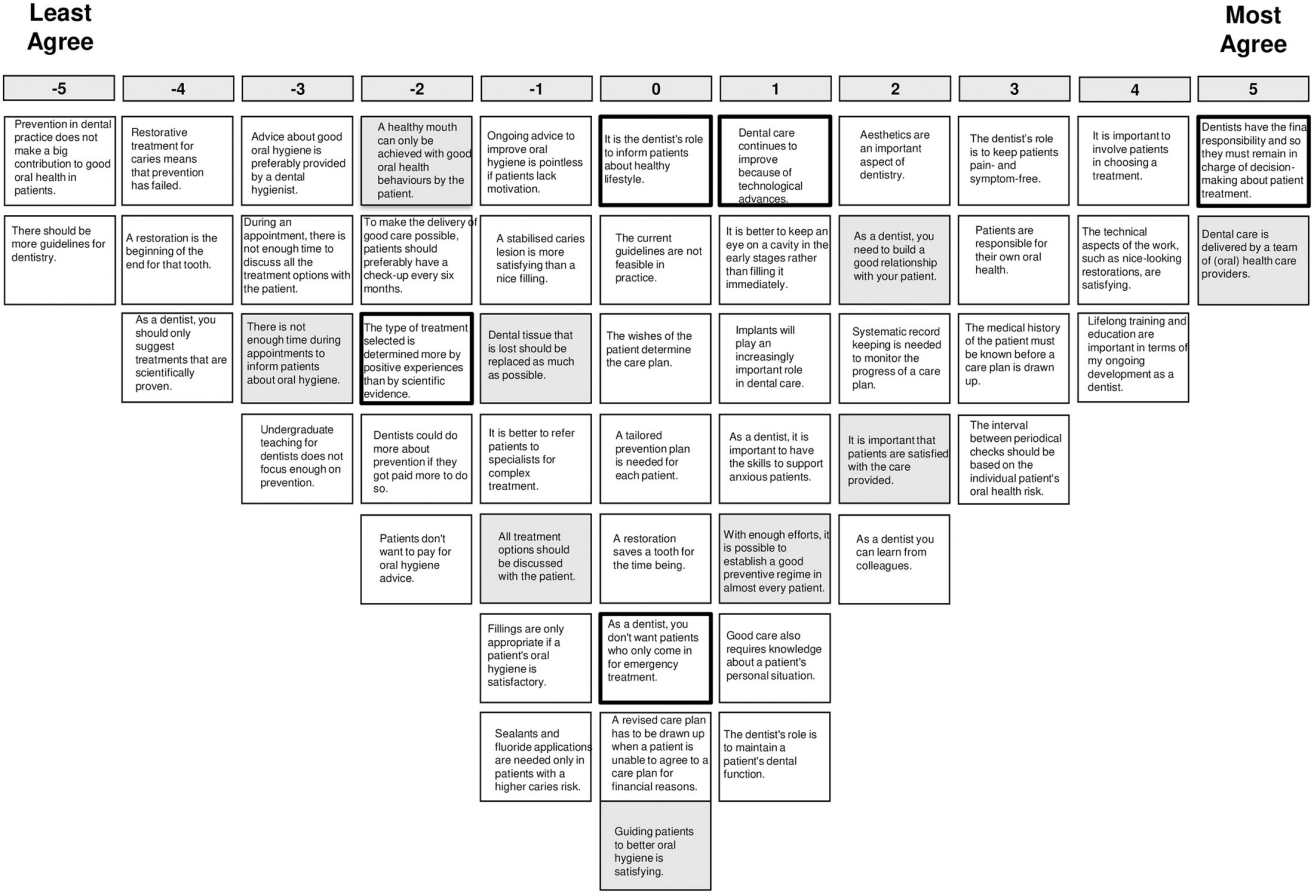
Score sheet for viewpoint 3. Note: Distinguishing statements (p<0.05) are shown in black outlined boxes, distinguishing statements (p<0.001) are shown in grey boxes.

**Fig 5:**
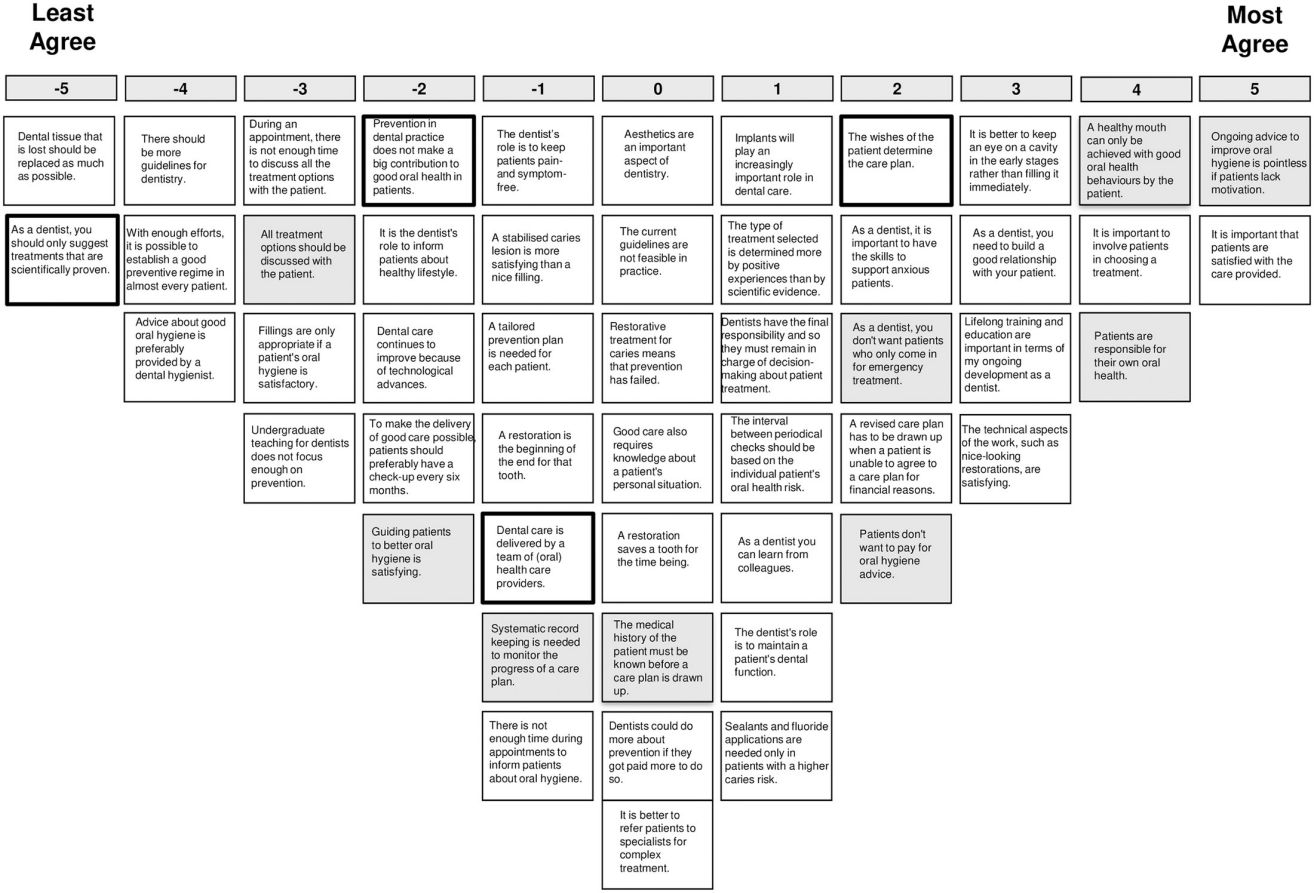
Score sheet for viewpoint 4. Note: Distinguishing statements (p<0.05) are shown in black outlined boxes, distinguishing statements (p<0.001) are shown in grey boxes.

**Fig 6:**
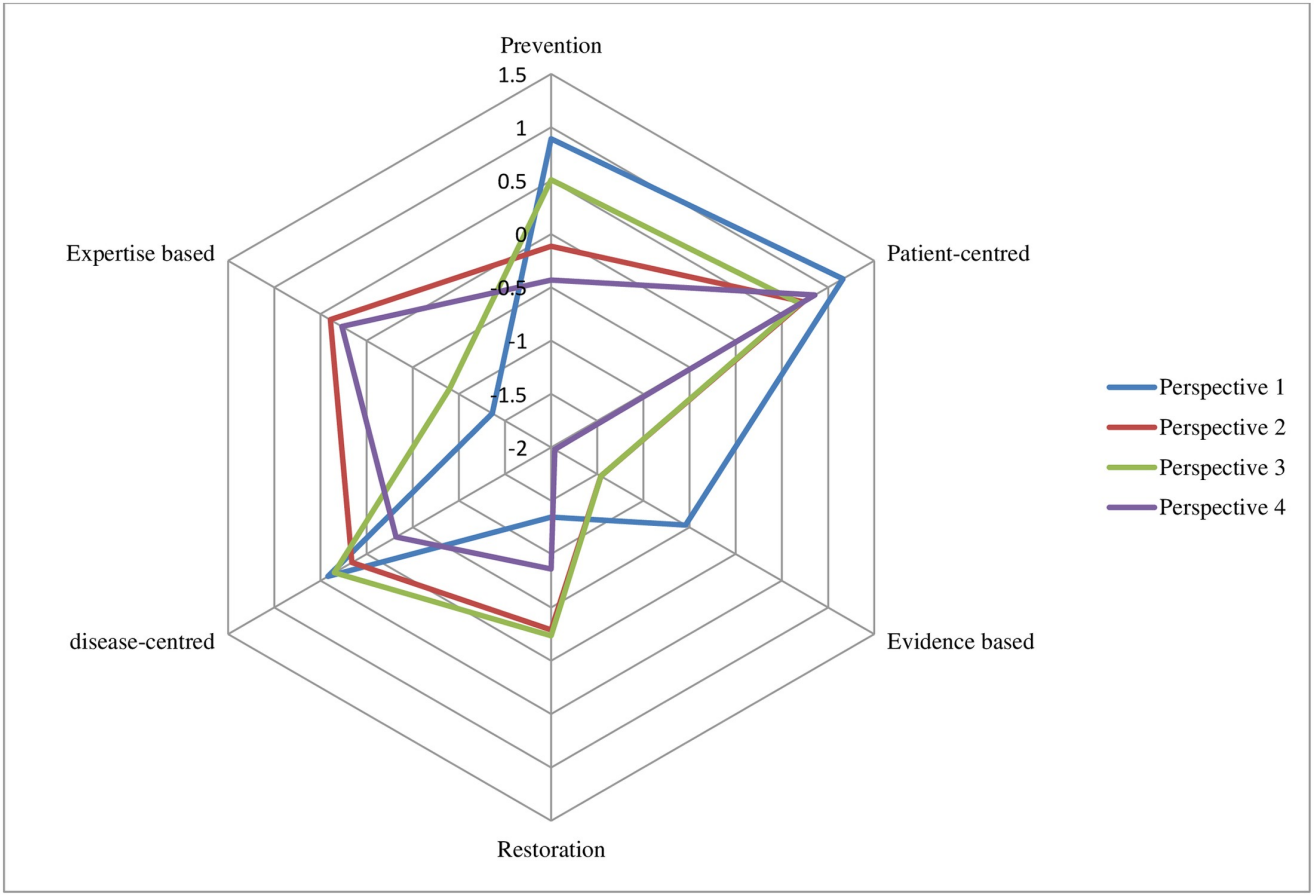
Mean Z-score Q-statements visualized amongst the domains: Prevention-restoration, patient- disease-centered and expertise based -evidence based. **Mean z-score of Q-statements listed in** Table 2 Prevention statements: no. 1, 14, 15 and 20 Restoration statements: no. 9, 19 and 28 Patient-centered statements: no. 10, 13, 17, 21, 29, 31, 37 and 38 disease-centered statements: no. 2, 22 and 47.Evidence-based statement: no. 50 Expertise-based statement: no. 24.

The analysis lead to the choice for a 4-factor solution. Adjacent factor solutions were also inspected, but the 4-factor solution provided the most distinct and interpretable and comprehensive set of factors, explaining 47% of the variance. Since the domains explored in this study are not counterpart of each other, any GDP’s individual perspective will not necessarily coincide with one of these four perspectives, but may exhibit elements from some or all of the perspectives to a different degree. Table 2 shows the idealized sorts of the four factors, as well as the distinguishing statements. Figs 2–5 show the idealized sorts of the four perspectives. The interpretations of the factors are described below and illustrated with citations from the explanations GDPs’ provided to their rankings of the statements (in *Italics*). Relevant statement numbers, as shown in Table 2, are referred to in parentheses. Fig 6 show the four perspectives according to the three key domains: i.) restorative—preventative OHC, ii.) disease-centred—patient-centred and iii.) expertise-based—evidence-based.

**Table 2:** Idealized sorting of the 50 statements for the full sample.

Statement		Attitude 1	Attitude 2	Attitude 3	Attitude 4
1	Prevention in dental practice does not make a big contribution to good oral health in patients.	-5	-3*	-5	-2*
2	The dentist’s role is to keep patients pain- and symptom-free.	3	0	3	-1
3	There should be more guidelines for dentistry.	-1**	-5**	-5	-4
4	It is the dentist’s role to inform patients about healthy lifestyle.	2**	-2	0*	-2
5	Aesthetics are an important aspect of dentistry.	-1	2	2	0
6	Dental care continues to improve because of technological advances.	-1	0*	1*	-2
7	A healthy mouth can only be achieved with good oral health behaviors by the patient.	2*	1*	-2**	4**
8	The current guidelines are not feasible in practice.	-4**	-1	0	0
9	It is better to keep an eye on a cavity in the early stages rather than filling it immediately.	5	1	1	3
10	It is important to involve patients in choosing a treatment.	5	3	4	4
11	Implants will play an increasingly important role in dental care.	-3**	-1**	1	1
12	Ongoing advice to improve oral hygiene is pointless if patients lack motivation.	-2	-2	-1	5**
13	The wishes of the patient determine the care plan.	4**	0	0	2*
14	A stabilized caries lesion is more satisfying than a nice filling.	1**	0	-1	-1
15	A tailored prevention plan is needed for each patient.	2**	0	0	-1
16	Restorative treatment for caries means that prevention has failed.	-1	-4	-4	0
17	As a dentist, it is important to have the skills to support anxious patients.	1	-1*	1	2
18	Patients are responsible for their own oral health.	0**	2	3	4**
19	The technical aspects of the work, such as nice-looking restorations, are satisfying.	-1**	2	4	3
20	With enough efforts, it is possible to establish a good preventive regime in almost every patient.	0**	-4	1**	-4
21	Good care also requires knowledge about a patient’s personal situation.	1	-1	1	0
22	To make the delivery of good care possible, patients should preferably have a check-up every six months.	-2	-1*	-2	-2
23	Advice about good oral hygiene is preferably provided by a dental hygienist.	-3	-2	-3	-4
24	The type of treatment selected is determined more by positive experiences than by scientific evidence.	-4*	1	-2*	1
25	A restoration saves a tooth for the time being.	-2**	0	0	0
26	Lifelong training and education are important in terms of my ongoing development as a dentist.	3	4	4	3
27	During an appointment, there is not enough time to discuss all the treatment options with the patient.	-3	-2	-3	-3
28	Dental tissue that is lost should be replaced as much as possible.	-5	-3**	-1**	-5
29	As a dentist, you need to build a good relationship with your patient.	4	4	2**	3
30	A restoration is the beginning of the end for that tooth.	-1	-5	-4	-1
31	The medical history of the patient must be known before a care plan is drawn up.	2	3	3	0**
32	Dentists have the final responsibility and so they must remain in charge of decision-making about patient treatment.	0	3*	5*	1
33	As a dentist, you don’t want patients who only come in for emergency treatment.	0*	-3**	0*	2**
34	Dental care is delivered by a team of (oral) health care providers.	1	1	5**	-1*
35	Systematic record keeping is needed to monitor the progress of a care plan.	1	1	2	-1**
36	Dentists could do more about prevention if they got paid more to do so.	-1	1*	-2	0
37	The interval between periodical checks should be based on the individual patient’s oral health risk.	3	1	3	1
38	It is important that patients are satisfied with the care provided.	4	5	2**	5
39	It is better to refer patients to specialists for complex treatment.	0	0	-1	0
40	A revised care plan has to be drawn up when a patient is unable to agree to a care plan for financial reasons.	0	2	0	2
41	As a dentist you can learn from colleagues.	0	5**	2	1
42	All treatment options should be discussed with the patient.	1**	3**	-1**	-3**
43	Fillings are only appropriate if a patient’s oral hygiene is satisfactory.	0*	-3	-1	-3
44	There is not enough time during appointments to inform patients about oral hygiene.	-4**	-1	-3**	-1
45	Patients don’t want to pay for oral hygiene advice.	-2	0**	-2	2**
46	The dentist’s role is to maintain a patient’s dental function.	3	2	1	1
47	Sealants and fluoride applications are needed only in patients with a higher caries risk.	1	-1	-1	1
48	Guiding patients to better oral hygiene is satisfying.	2**	4**	0**	-2**
49	Undergraduate teaching for dentists does not focus enough on prevention.	-3	-2	-3	-3
50	As a dentist, you should only suggest treatments that are scientifically proven.	-2**	-4	-4	-5*

* distinguishing statements p<0.05

** distinguishing statements p<0.01

### Perspective 1: The patient-focused dentist who values prevention

The patient has a central position within this perspective on OHC: “*Everything in dentistry is about the patient*, *not the dentist*”. Involving the patient in decision-making is considered important (st.10, +5; i.e., statement 10 received score +5; see Fig 2 for the idealized sort of perspective 1). Shared decision-making is perceived as a way to improve the quality of OHC: “*When a treatment is chosen in consultation with the patient*, *the chance of success is higher*”. The wishes of the patient should be in the lead when determining the care plan (st.10, +5; st.13, +4): “*The patient remains the boss over their own body*”. Having a good relationship with the patients is valued, and it is viewed as a task of the dentist to build a good relationship with the patient (st.29, +4; st.38, +4). Furthermore, these GDPs distinguish themselves from those with the other perspectives in that care should be tailored to the needs of individual patient (statements 13, 15 and 37; scores 4, 2 and 3), instead of simply following protocols or guidelines in all cases (st.37, +3): “*There is no need to have all patients come for a check-up every 6 months*, *especially not for adults with a stable dentition and periodontium*”.

The GDPs with this perspective feel responsible to keep their patients’ teeth functional and pain free (st.2, +3; st.46, +3). Consequently, monitoring rather than intervention is valued; the GDPs feel that restoration is not always the only or immediate step in fixing a problem (st.9, +5; st.28, -5). In contrast to GDPs with perspective 2, they are convinced of the usefulness of prevention and regard it essential for good oral health (st.1, -5; st.11, -3): “*Prevention is the start of everything*, *there is no healthy mouth without prevention*”. Additionally, they feel that time is not (and should not) be a constraint in providing advice or involving a patient in decision-making (st.27, -3; st.44, -4): “*It can never be the case that there is insufficient time to explain the goal of care*”, “*You can make time*”. They feel they have the skills to provide such care and feel that prevention is sufficiently covered during their dental education (st.49, -3). Also, they do not agree that patients are not willing to pay for oral hygiene advice (st.45, -2). Distinguishing for this perspective is that the GDPs not only find prevention of oral diseases important, but also consider it their role to inform and advice on a healthy lifestyle in general (st.4, + 2): “*What the patients eats and drinks mainly determines the health of the mouth*”.

Compared to the other perspectives, these GDPs attach most value on evidence-based care and do not consider own experience superior to scientific evidence (st.8, -4; st.24, -4). Accordingly, they are most positive about the feasibility of current guidelines (st.8, -4) and least opposed to more guidelines (st.3, -1).

### Perspective 2: The outcome-oriented dentist who values learning from colleagues

GDPs with this perspective value a good patient-practitioner relationship with mutual trust and patients being satisfied with the care they receive (st.29, +4; 38; +5; see Fig 3 for the idealized sort of perspective 2): *“Without a good relationship there cannot be trust and the results will not be optimal”*. They feel it is important to involve patients in decision-making (st.10, +3) and to discuss all treatment options with them (st.42, +3), but unlike those with the previous view, the GDP in the end should be in charge of the treatment choice (st.32, +3).

GDPs with this perspective take great value in keeping developing oneself as a dentist by continued training and learning from colleagues (st.26, +4; st.41, +5): “*Lifelong training is essential after a limited education*”; “*Colleagues broaden my view on the dental situation and dentistry instead of my own narrow view*”.

These GDPs consider guiding patients towards better oral health as a primary goal (st.48, + 4; st.32, +3). But in this perspective it is less relevant whether this is accomplished by prevention or treatment. Restoration is not perceived as failure of prevention (st.16, -4; st.30, -5), and they do not hold great expectations about prevention—they believe it is not possible for every patient (st.20, -4). This perhaps also explains why, compared with the other perspectives, they do not find it a problem seeing patients who only visit the GDP when they have a dental emergency (st.33; -3).

The GDPs with this perspective are not in favour of more guidelines; indeed, of the four perspectives these GDPs feel strongest about this. They feel constrained and already overwhelmed by current guidelines (st.3, -5): “*Everything is being pushed into guidelines*, *this is too much*”. Moreover, these GDPs do not think that guidelines should always be followed, nor do they feel that only scientifically proven treatments should be suggested (st.50; -4).

### Perspective 3: The team player with ultimate care responsibility

These GDPs see dental care as the effort of a team of health care providers (st.34, +5; see Fig 4 for the idealized sort of perspective 3): *“You cannot reach quality only by yourself*. *Quality of care is reached with a team*”. In comparison to the other perspectives, these GDPs are less concerned with patient satisfaction or the relationship with the patient (st.29, +2; st.38, + 2). After all, the dentist is responsible for the care delivered to patients and should therefore remain in charge of treatments decisions (st.2, +3; st.32, +5): *“Ultimately it is the dentists who is the most educated*, *has the most insight and has the knowledge”*. To secure this, continued training and education is considered crucial (st.26, +4).

Restoration and prevention are both seen as useful and potentially effective ways to improve oral health (st.1, -5; st.16, -4; st.30, -4), but, most of all GDPs, those with this view derive satisfaction from the technical aspects of care delivery (st.19, +4).

GDPs with this perspective on OHC also feel that evidence-based care has its limitations. They feel that only providing evidence-based care is impossible (st.50, -4): “*Providing only evidence-based care means that you will not get any further*”. More guidelines are therefore also considered to be restrictive and not helpful (st.3, -5): “*With more guidelines there will be more stress amongst the staff”; “It is already difficult to cope with the current guidelines and protocols*”.

### Perspective 4: The dentist who considers oral health the responsibility of the patient

This perspective on OHC is most of all characterized by the view that patients are considered to be responsible for their own oral health (st.18, +4; see Fig 5 for the idealized sort of perspective 4): “*How you behave and what choices you make will always be your own responsibility”*. A healthy mouth can only be achieved with good oral health behaviour by the patient (st.7, +4). The GDPs within this perspective find it least of all the other perspectives their role to keep patients pain and symptom free (st.2, -1). However, within this perspective satisfaction of the patient with the care provided is important (st.38, +5). Therefore, it is considered important to involve the patients in decision-making (st.10, +4) and to include their wishes in determining the care plan (st.13, +2). However, according to these GDPs this does not mean that all treatment options should be discussed with the patient (st.42, -3). “*Sharing all treatment options does not mean they are reasonable options*”.

The GDPs within this perspective seem least positive about prevention (st.1, -2). Guiding patients to better oral hygiene is not being considered to be satisfying (st.48, -2), nor do they not think it is their role to inform patients about a healthy lifestyle (st.4, -2). Ongoing advice to improve oral hygiene is pointless if patients lack motivation (st.12, +5).” *You will not get there with only the efforts of the dentist*, *the patient has to put their own effort in it*”. In their view, it is not possible to establish a good preventive regime in almost every patient, even with enough efforts (st.20, -4). In addition, these GDPs feel there is sufficient focus on prevention in undergraduate teaching for dentists (st.49, -3), and they are quite ambivalent about dentists possibly doing more about prevention if they got paid more to do so (st.36,0). Patients themselves also don’t want to pay for oral hygiene advice (st.45, +2). However, the GDPs feel that restoration is not always the only or immediate step in fixing a problem (st.9, +3; st.28, -5). When possible, monitoring is preferred from filling a cavity (st.9, +3). GDPs with this perspective prefer not to have patients that only visit the dental practice for emergency treatment (st.33, 2).

The GDPs with this perspective also do not attach importance to the evidence-base (st.50, -5): “*Evidence based care is not achievable*, *often the evidence is contradictory*”. Similar to the other perspectives, these GDPs feel that there should not be more guidelines (st.3, -4).

## Discussion

This study explored perspectives on OHC amongst GDPs in the Netherlands. On the basis of this study, four distinct perspectives were identified: 1) the patient-focused dentist who values prevention, 2) the outcome-oriented dentist who values learning from colleagues, 3) the team player with ultimate care responsibility and 4) the dentist who considers oral health the responsibility of the patient. Apart from their distinctive features, these perspectives also have some common elements (see Fig 6). GDPs holding perspectives 1, 2 and 4 considered the patient-practitioner relationship important in care delivery, whereas GDPs with perspective 3 put lesser value on this. The perspectives 1 and 4 showed the strongest belief in prevention, whereas perspectives 2 and 3 did not necessarily prioritize prevention ahead of restoration. Evidence-based OHC delivery was a preference over expertise only in perspective 1.

Findings by Honey et al. (2013) suggest that healthcare professionals hold perspectives that may influence how they interact with patients [19]. This study found that this also holds for GDPs. For example, GDPs that value sharing information on all treatment options (perspective 2) may have different consultations with patients regarding care decisions in comparison to GDPs that do not feel it is relevant to discuss all options with patients (perspective 4). As the perspectives amongst GDPs on shared decision making and evidence-based care differ, this will consequently lead to variation in OHC. As an example, patients cannot opt for a treatment that is not offered to them. Joseph-Williams et al. (2014) found that in the implementation of shared decision-making that practitioners feel they already involve patients in decisions, while this is not always the case [20]. Furthermore, a study conducted in the United Kingdom showed that patients perceived their role in decision making as passive whilst they preferred a collaborative decisional role with their GDP [21].

Tailoring care according to the current changes in dentistry towards patient-centred, prevention and evidence-based care is not straightforward. Decisions are rarely based on knowledge explicitly retrieved from research findings, clinical guidelines or other sources of formal knowledge only [22,23]. Various other factors influence and shape the perspectives GDPs have on their roles and tasks in care delivery, and consequently on the decisions they make with or for patients in oral healthcare delivery. Gabbay and le May argued that clinicians tend to base their decisions on “mindlines”, referring to internalised and collectively reinforced tacit guidelines which are mainly based on the interactions with, opinion leaders, patients, pharmaceutical company representatives and others [23]. These mindlines are thus created in social processes, through discourse, influenced by cultural and historic factors [24], and strongly rely on individual preferences and implicit professional norms and values. Berwick speaks of a collision of norms and values amongst care providers due to clashes between the ethos of professionalism and the ethos of markets and accountability [22]. The GDPs holding perspective 4 seem to reflect the ethos of professional autonomy and trust, whilst those holding perspective 1 more closely reflect the views of believers in accountability, scrutiny, measurement and markets.

This study corroborates earlier research that showed a generally positive attitude among GDPs towards preventive care [25]. The participants generally did not agree with critical statements about prevention, for example statement 1: “Prevention in dental practice does not make a big contribution to good oral health in patients”. However, the four perspectives showed variation in how much and to whom preventive care should be provided. Where GDPs with perspective 4 consider providing oral hygiene advice pointless when patients are unmotivated, GDPs with the other perspectives appeared less convinced of this. Furthermore, GDPs might have strong feelings about patients receiving oral hygiene advice, but are sceptical about lifestyle advice (e.g. smoking cessation, dietary advice). As lifestyle advice goes beyond the oral cavity, some GDPs might consider lifestyle advice not part of their tasks or their responsibility. Finally, some GDPs might consider prevention not to be part of their tasks because they refer to prevention assistants or dental nurses to provide preventive treatments and advice. The strength of using Q-methodology for our study goals, is that it combines the rigor and transparency of quantitative methods with the richness and depth of qualitative methods. Perspectives of GDPs have previously been measured by means of questionnaires using Likert-type scales or questionnaires using ratings [26,27]. These methods show a tendency among respondents to agree or indicate a positive connotation with the questions, and to limit the scope of their response to the topics presented to them by the researcher. Furthermore, analysing the questionnaires delivers an overall-score that limits a person to one perspective [28]. In contrast, Q-methodology combines the systematic rigor of quantitative methods with the depth and openness of qualitative methods [29].

There are several limitations to this study. Efforts have been made to develop a complete Q-set, based on literature and interviews, that includes the important aspects of OHC on which GDPs gave their perspectives. Pilot testing of the statements and use within this study did not give any reason for doubt about the relevance and completeness of the statements. However, it would be relevant to test this when repeating the study in a different context. Q-methodology relies on a theoretical selection of respondents to explore a range and variety of perspectives. Within this study a broad representation of GDPs was involved to reveal the range of perspectives. A different recruited sample is therefore expected to reveal similar perspectives when using the same theoretical structure. However, it is impossible to exclude the possibility that a perspective has been missed; further research would be needed to confirm or refute this.

This study included the opinions of GDPs in the Netherlands only. The generalizability might be limited as perspectives on OHC might be different in different countries, because of differences in financing and insuring OHC systems and different curricula and culture in different dental schools. It would therefore be interesting to repeat this study in other settings and to compare the findings, and so to learn whether and how differences in education or health care systems are related to perspectives on OHC. But for the Netherlands these perspectives can be considered to be representative of the range and variety of perspectives among GDPs. Inherently to the Q-methodology approach, is that no conclusions can be drawn about the proportion of the GDPs that hold these viewpoints, or on associations of the perspectives with characteristics of GDPs, the actual care provided, OHC systems, or other variables of interest. This information could be obtained with a follow-up survey approach, and would be a valuable extension of the current study for assessing whether and how different perspectives may affect care delivery.

This is one of the first studies that used Q-methodology in dentistry. Vermaire et al. (2010) used it to study the parental attitudes towards oral health and the dental care of their children [30], Trubey et al. (2013) studied attitudes of community dental service staff towards a daily supervised tooth brushing program [31], and there are two studies on patient and parental attitudes to orthodontic treatments [32,33]. A comparable study by Eijsink, et al. (2018) demonstrated variation in the extent to which caregivers feel responsible for OHC of institutionalized persons with moderate intellectual disability [25]. This study provides insight into the perspectives of GDPs and is not focused on one specific area of OHC, but looks at OHC as a whole. Insight into the different perspectives GDPs hold can promote awareness among stakeholders about this diversity in perspectives and the possibly associated variation in care delivery.

It is important to note that the perspectives presented here are not meant to label GDPs as a “good” or “bad” dentist. The perspectives reveal that GDPs hold different perspectives that may lead to different interactions with patients and perhaps to different care decisions and outcomes. Awareness of these differences can be used as a starting point for quality improvement strategies. Further, the perspectives provide GDPs with insight into their own conscious and perhaps unconscious preferences which can be used to evaluate and improve their OHC delivery, and stimulate discussions with peers about improving OHC practice.

## Conclusion

This study provided new insights into the perspectives among GDPs concerning OHC delivery. It demonstrates that GDPs should not be regarded as a homogenous group. It seems advisable to acknowledge and account for this heterogeneity when developing and implementing future quality improvement strategies. Revealing the variation in GDPs’ perspectives can contribute to creating awareness amongst GDPs about their own OHC delivery and that of their colleagues. Awareness amongst GDPs about their own attitudes and preferences might help GDPs align their perspectives with those of their patients. Theoretically, it could help inform the quality of care improvement strategies adopted by individual GDPs. Whether perspective-informed quality improvement strategies will result in an improved care delivery from either an individual or a population-based perspective is a matter for further research.

## Supporting information

S1 Table Q-statements, categorized according to themes and domains. (DOCX)
