# Correction: Perspectives of general dental practitioners on preventive, patient-centred, and evidence-based oral healthcare—A Q-methodology study

**DOI:** 10.1371/journal.pone.0223458

**Published:** 2019-10-01

**Authors:** Fatiha Baâdoudi, Job van Exel, Fatima M. Ali, Neal Maskrey, Geert J. M. G. van der Heijden, Denise Duijster

The second author’s name appears incorrectly. The correct name is Job van Exel. The correct citation is: Baâdoudi F, van Exel J, Ali FM, Maskrey N, van der Heijden GJMG, Duijster D (2019) Perspectives of general dental practitioners on preventive, patient-centred, and evidence-based oral healthcare—A Q-methodology study. PLoS ONE 14(8): e0219931. https://doi.org/10.1371/journal.pone.0219931.
