# Supplementary material for: Perspectives of general dental practitioners on preventive, patient-centred, and evidence-based oral healthcare—A Q-methodology study
Source: PLoS One. 2019 Aug 20;14(8):e0219931. doi: 10.1371/journal.pone.0219931 (PMC6701752; doi:10.1371/journal.pone.0219931)
Supplement: S1 Table — (DOCX) [file pone.0219931.s001.docx]

**S1 Table. Q-statements, categorized according to themes and domains.**

| **Prevention - restoration domain** |
| --- |
| **Appreciation of prevention** |
| Prevention in dental practice does not make a big contribution to good oral health in patients. |
| Restorative treatment for caries means that prevention has failed. |
| Fillings are only appropriate if a patient's oral hygiene is satisfactory. |
| **Responsibility for prevention** |
| It is the dentist's role to inform patients about healthy lifestyle. |
| Advice about good oral hygiene is preferably provided by a dental hygienist. |
| A healthy mouth can only be achieved with good oral health behaviours by the patient. |
| Patients are responsible for their own oral health. |
| **Dentists' own effectiveness with respect to prevention** |
| With enough efforts, it is possible to establish a good preventive regime in almost every patient. |
| Ongoing advice to improve oral hygiene is pointless if patients lack motivation. |
| **Barriers to prevention** |
| Dentists could do more about prevention if they got paid more to do so. |
| There is not enough time during appointments to inform patients about oral hygiene. |
| Patients don't want to pay for oral hygiene advice. |
| **Personalised prevention** |
| A tailored prevention plan is needed for each patient. |
| Sealants and fluoride applications are needed only in patients with a higher caries risk. |
| **Dentist satisfaction** |
| A stabilised caries lesion is more satisfying than a nice filling. |
| Guiding patients to better oral hygiene is satisfying. |
| The technical aspects of the work, such as nice-looking restorations, are satisfying. |
| **Appreciation of restoration** |
| A restoration saves a tooth for the time being. |
| A restoration is the beginning of the end for that tooth. |
| Dental care continues to improve because of technological advances. |
| Dental tissue that is lost should be replaced as much as possible. |
| Implants will play an increasingly important role in dental care. |
| **Monitoring** |
| It is better to keep an eye on a cavity in the early stages rather than filling it immediately. |
| Systematic record keeping is needed to monitor the progress of a care plan. |
| **Patient-based – disease-based domain** |
| **Personalised care** |
| The interval between periodical checks should be based on the individual patient's oral health risk. |
| To make the delivery of good care possible, patients should preferably have a check-up every six months. |
| As a dentist, it is important to have the skills to support anxious patients. |
| **Information about patient status** |
| As a dentist, you need to build a good relationship with your patient. |
| The medical history of the patient must be known before a care plan is drawn up. |
| Good care also requires knowledge about a patient's personal situation. |
| **Shared decision-making** |
| It is important to involve patients in choosing a treatment. |
| The wishes of the patient determine the care plan. |
| Dentists have the final responsibility and so they must remain in charge of decision-making about patient treatment. |
| As a dentist, you don't want patients who only come in for emergency treatment. |
| A revised care plan has to be drawn up when a patient is unable to agree to a care plan for financial reasons. |
| All treatment options should be discussed with the patient. |
| During an appointment, there is not enough time to discuss all the treatment options with the patient. |
| **Duties of the dentist** |
| The dentist's role is to maintain a patient's dental function. |
| Aesthetics are an important aspect of dentistry. |
| The dentist's role is to keep patients pain- and symptom-free. |
| Dental care is delivered by a team of (oral) health care providers. |
| **Patient satisfaction** |
| It is important that patients are satisfied with the care provided. |
| **Evidence-based – experience-based domain** |
| **Evidence** |
| As a dentist, you should only suggest treatments that are scientifically proven. |
| The current guidelines are not feasible in practice. |
| There should be more guidelines for dentistry. |
| **Training and continuous education** |
| Lifelong training and education are important in terms of my ongoing development as a dentist. |
| As a dentist you can learn from colleagues. |
| Undergraduate teaching for dentists does not focus enough on prevention. |
| **Expertise** |
| It is better to refer patients to specialists for complex treatment. |
| The type of treatment selected is determined more by positive experiences than by scientific evidence. |
